# The Impact of Participant Characteristics on Use and Satisfaction of a Web-Based Computer-Tailored Chronic Obstructive Pulmonary Disease Self-Management Intervention: A Process Evaluation

**DOI:** 10.2196//formative.6585

**Published:** 2017-08-21

**Authors:** Viola Voncken-Brewster, Mylène Amoureus, Hein de Vries, Zsolt Nagykaldi, Bjorn Winkens, Trudy van der Weijden, Huibert Tange

**Affiliations:** ^1^ Department of Family Medicine Care and Public Health Research Institute Maastricht University Medical Center Maastricht Netherlands; ^2^ Institute of Health Policy and Management Erasmus University Rotterdam Rotterdam Netherlands; ^3^ Department of Health Promotion Care and Public Health Research Institute Maastricht University Medical Center Maastricht Netherlands; ^4^ Department of Family and Preventive Medicine University of Oklahoma Health Sciences Center University of Oklahoma Oklahoma, OK United States; ^5^ Department of Methodology and Statistics Care and Public Health Research Institute Maastricht University Medical Center Maastricht Netherlands

**Keywords:** Internet intervention, computer tailoring, application use, participant characteristics, COPD, self-management, behavior change, process evaluation

## Abstract

**Background:**

A randomized controlled trial (RCT) showed that a Web-based computer-tailored self-management intervention for people with or at risk for chronic obstructive pulmonary disease (COPD) did not have a significant treatment effect. Process evaluation measures such as application use and satisfaction with the intervention can help understand these results.

**Objectives:**

The aim of this paper is to uncover reasons for suboptimal application use, evaluate satisfaction with the intervention, and investigate which participant characteristics predict application use and user satisfaction.

**Methods:**

Participants were recruited through 2 different channels: an online panel and general practice. The intervention group received the intervention, which consisted of 2 modules (smoking cessation and physical activity). The control group received no intervention. The study employed a mixed methods design. Quantitative and qualitative data were gathered assessing participant characteristics, application use, reasons for not using the application, and satisfaction with the intervention.

**Results:**

The RCT included 1325 participants. The proportion of individuals who participated was significantly higher in the online group (4072/6844, 59.5%) compared to the general practice group (43/335, 12.8%) (*P*<.001). Application use was low. Of all participants in the intervention group, 52.9% (348/658) initiated use of one or both modules, 36.0% (237/658) completed an intervention component (prolonged use), and 16.6% (109/658) revisited one of the modules after completing an intervention component (sustained use). Older age, established diagnosis of COPD, or experiencing breathlessness predicted sustained use. Participant satisfaction with the 2 modules was 6.7 (SD 1.6) on a scale from 0 to 10. The interviews revealed that a computer application was believed not to be sufficient and the help of a health care professional was necessary. Participants with a greater intention to change were more satisfied with the application.

**Conclusions:**

The application was not used sufficiently. Study materials should be further tailored to younger individuals, those at risk for COPD, and those who do not experience breathlessness in order to increase sustained use among them. Involvement of a health care professional could improve satisfaction with the intervention and potentially increase engagement with the intervention materials. However, to make this possible, recruitment in general practice needs to be improved. Recommendations are made for improving the study design, strengthening the intervention (eg, practice facilitation), and linking the computer application to interaction with a health care provider.

## Introduction

Chronic obstructive pulmonary disease (COPD) is a highly prevalent disease characterized by airway obstruction that is not fully reversible [1]. In order to decelerate the progression of the disease, interventions focusing on self-management and behavior modification such as smoking cessation and physical activity, are considered important [2,3]. Multiple COPD self-management interventions have been developed; most of these interventions include helping patients with physical activity and/or smoking behavior, but their effectiveness remains uncertain [4].

Supporting patients in improving smoking and physical activity behaviors can be achieved by using information and communication technology [5,6]. Several tools have been developed for COPD patients. For instance, one study found short-term effects of an Internet-mediated, pedometer-based walking program on daily step count and health-related quality of life [7]. However, no long-term effects were found [8]. Another study showed that a mobile activity monitoring and feedback tool for COPD and type 2 diabetes patients effectively increased physical activity when combined with counseling [9]. In the MasterYourBreath project, we developed a COPD self-management intervention using computer-tailored technology to improve smoking behavior and levels of physical activity. Computer-tailored technology makes it possible to provide individuals with computer-generated personally relevant health promotion information at their own home [5]. Relevant feedback can be given by tailoring messages to participant characteristics, which has been found to increase participant attention, appreciation, and thorough processing of information [10-12]. Computer-tailored interventions have often been used to prevent disease in the general population [13-15] and have shown to effectively aid smoking cessation and increase physical activity [16-17]. However, the results of a randomized controlled trial (RCT) testing the MasterYourBreath intervention showed no significant treatment effect of the intervention on behavior and clinical outcomes in COPD patients and people at risk for COPD [18].

The lack of treatment effect could be explained by a number of reasons related to the intervention, including suboptimal application design [19], recruitment problems [20] and inadequate use of the application [20-23]. Most of these potential problems were already detected and considered during the preparation of the RCT. For example, we had improved the user interface design during a usability study [24]. We also evaluated the feasibility of integrating the application into an existing disease management approach [25] by conducting a pilot study [26]. In the pilot study, participants were recruited in family practices by mediation of the practice nurse, which did not result in the required number of respondents. To improve study participation during the RCT, we broadened the recruitment strategy by including people at risk for COPD in addition to diagnosed COPD patients, inviting patients from general practices by mail, and by recruiting people from an online panel. This strategy improved the reach of our target population, but hindered our plans for integrating the MasterYourBreath intervention into primary care [18]. A problem we were not able to solve adequately was the suboptimal use of the application. We included several evidence-based measures to promote application use during the RCT [18] based on results of the pilot study [26]. However, application use was still low [18], which could be a potential explanation for the nonsignificant effect on primary outcomes in the RCT. Protocol analyses including only participants who used main components of the intervention showed no significant effects on smoking cessation and physical activity, possibly due to the limited sample size and thus decreased power of the study. However, a trend was found for an increased effect size for smoking cessation and physical activity, which was related to the number of completed intervention components [18]. We were not able to determine the threshold for sufficient application use, but the completion of more components was associated with an increased treatment effect.

Considering application use, it is important to understand which intervention characteristics and participant characteristics are associated with the adoption of the intervention materials, as explained in the diffusion of innovation theory [27]. It is important to know which intervention characteristics were appreciated, who visited and revisited the application, and which participant characteristics were associated with satisfaction in order to explain use rates and develop better strategies to increase application use. To our knowledge, no studies have been conducted investigating which participant characteristics predict the use and satisfaction of online health promotion interventions in COPD patients. Studies focusing on online health promotion applications in other target populations show mixed results [28-30]. For example, Brouwer et al [28] found that younger women with a medium-to-high education level were more likely to use behavior modules. Stretcher et al [30] found the same for gender and education level but the opposite for age, as an older age was positively associated with application use. The study of Schneider at al [29] also showed a positive impact of older age on module use. However, contrary to the other studies, this study found that men were more likely to use the modules. Another interesting finding was that participants with a relatively unhealthy lifestyle and low income were more likely to initiate a module, but they were less likely to complete a module.

In this paper, we report the results of a process evaluation, conducted in conjunction with the RCT of the MasterYourBreath intervention, in order to examine possible reasons for insufficient use of the application and to explore user satisfaction. The evaluation focused on suboptimal application use and user satisfaction in general and the influence of participant characteristics on application use and user satisfaction.

## Methods

### Study Design

The process evaluation study was conducted as part of an RCT examining the effect of a computer-tailored self-management intervention targeting smoking cessation and level of physical activity [18,31]. A mixed methods study design was employed using quantitative and qualitative data complementarily. The study applied a triangulation design model [32], in which the quantitative and qualitative data were integrated during the interpretation phase to understand the reasons for suboptimal use and to evaluate satisfaction. Data collection started in May 2012 and ended in July 2013, concurrent with the data collection of the RCT. The study was approved by the Medical Ethical Committee of Maastricht University Medical Center (METC 12-4-033) as part of the RCT.

### Recruitment

Adults between 40 and 70 years of age were eligible to participate if they were diagnosed with COPD or were at moderate or high risk for COPD, were proficient in Dutch, had access to the Internet and had basic computer skills. The Respiratory Health Screening Questionnaire (RHSQ) [33] was administered to determine if individuals were at moderate or high risk for COPD. Participants were recruited from 5 family practices that were involved in another study in which patients were screened for COPD by their general practitioner using the RHSQ [34] and from members of an existing Dutch online panel assembled by Flycatcher (www.flycatcher.eu), an International Organization for Standardization–certified institute for online research. Patients of the family practices received a paper invitation letter and were not compensated for the study. Members of the online panel received an invitation by email and were compensated with a small incentive equal to €2.55 (US $3) per completed questionnaire. A reminder was sent to those who did not reply to the study invitation. Participants were only compensated for completing baseline and follow-up questionnaires and not for using the MasterYourBreath application. All participants received a study information letter and completed an informed consent form before entering the study. Participants were randomly allocated to the intervention and control groups. Participants in the intervention group received the MasterYourBreath intervention. Participants in the control group received no intervention and did not have access to the website.

### MasterYourBreath Intervention

The intervention aimed to improve smoking cessation and physical activity by means of a Web-based application. One module was developed for each behavior, based on previously developed interventions [11,35,36] and adjusted for the target population. The I-change model [37,38] was used as the theoretical framework for this intervention. This model is the successor of the attitude-social influence-self-efficacy model [39] and incorporates several theoretical concepts from sociocognitive models such as the theory of planned behavior [40], social cognitive theory [41], transtheoretical model [42], health belief model [43], and implementation and goal-setting theories [44-45].

The 2 modules (smoking cessation and physical activity) consisted of 6 intervention components each: (1) health risk appraisal, (2) motivational beliefs, (3) social influence, (4) goal-setting and action plans, (5) self-efficacy in order to change behavior, and (6) self-efficacy in order to maintain behavior. Participants could switch between the smoking cessation and physical activity modules and choose to enter one or more intervention components at their preference [10]. Intervention components were available to be completed as often as participants chose over the course of the study.

**Figure 1:**
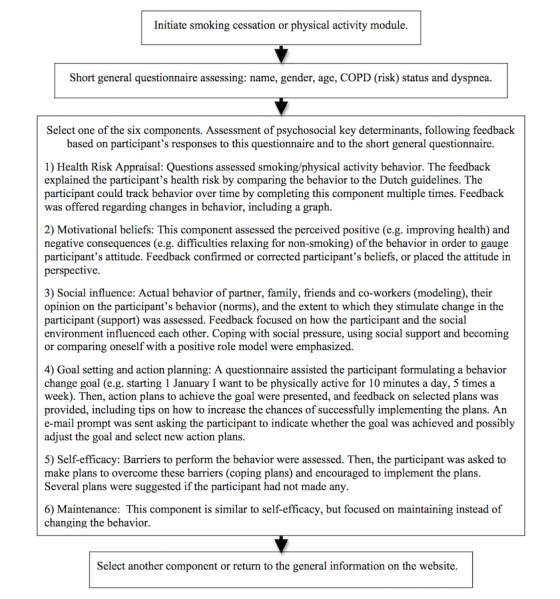
Overview of the main intervention content.

Each component provided participants with computer-generated tailored feedback based on participant responses to questionnaires. The feedback was personalized using participant names and tailored to participant characteristics including gender, age, COPD (risk) status, and level of disability. For example, feedback focused on stopping disease progression for COPD patients, while feedback for participants at risk for COPD focused on disease prevention. Feedback for COPD patients also acknowledged that COPD can limit their physical abilities, and feedback included suggestions to improve physical activity accordingly. Feedback was also tailored to behavior determinants based on psychosocial constructs. For example, barriers to quit smoking and plans to overcome these barriers were assessed and feedback was provided in order to increase participant self-efficacy [24]. In addition, participants could track their own behavior change and goal attainment, as the feedback compared previous responses to the most current responses. See Figure 1 for an overview of the main intervention content.

The behavior change modules for smoking cessation and physical activity were embedded in a website. The website offered participants information about the MasterYourBreath project, COPD, risk for COPD, smoking, and physical activity. The website also included nontailored self-management resources such as home exercise videos and hyperlinks to other informative websites. The website was not part of the core intervention content but was meant to attract participants and improve the user experience. Tailored feedback was kept as short as possible by referring to information and self-management resources on the website. The website was updated regularly with new information to maintain participant interest in the application [46-48].

Participants in the intervention group received an email invitation to use the application ad libitum for 6 months. They could access the application online with their personal account information, which was included in the email. If they did not use a behavior change module within 2 weeks after receiving the invitation, they were prompted by email. Another prompt was sent 2 weeks later if they did not respond to the first prompt. The 2-week time interval has shown to be optimal [49]. If participants had visited one of the two modules, prompts were sent every month to encourage revisits, so participants visiting both modules received a prompt approximately every 2 weeks. These prompts contained an option for participants to stop receiving future prompts. Prompts were tailored to COPD or individuals at risk for COPD and the selected behavior (smoking cessation or physical activity). Prompts included information to attract participants to the application—for example, by referring to new content on the website [49]. Participants who formulated concrete behavior-change goals received one email prompt 1 week after their goal was due. A more detailed description of the intervention can be found in the study protocol [31].

### Data Collection

#### Quantitative Data

A Web-based questionnaire was administered at baseline and after the 6-month intervention period. Demographic antecedents were gathered from an online database (online panel group) or as part of the baseline Web-based questionnaire (general practice group). Application use of each participant was monitored by the system.

#### Qualitative Data

The Web-based questionnaire also contained a comment section in which participants could voice their opinion about the application. The research team took field notes concerning the recruitment procedure and other communications with participants. Semistructured face-to-face interviews with 10 participants who used the MasterYourBreath application were conducted by an independent researcher (Mylène Amoureus). In order to create a heterogeneous sample, the interviewees were selected based on recruitment channel (online panel or general practice), age, gender, COPD (risk) status, education level, and smoking status. The interviews took place after the intervention period. During the interview, participants were asked to use the application in order to refresh their memory. Interviewees received a €25 (US $29) voucher.

### Measures

#### Participant Characteristics

Participant characteristics included personal, health status, and health behavior characteristics. Personal characteristics were gender, age, and education level (recoded as “low,” 1=primary school/basic vocational school; “medium,” 2=secondary vocational school/high school degree; and “high,” 3=higher professional degree/university degree). Health status characteristics were COPD status (coded as diagnosed with COPD or at risk for COPD) and dyspnea status, measured by the Medical Research Council (MRC) dyspnea score [50] (1 to 5, higher score means worse dyspnea). Health behavior characteristics included current smoking status (smoking/not smoking), level of physical activity assessed by the International Physical Activity Questionnaire–Short Form (IPAQ-SF) metabolic equivalent task (MET) minutes per week [51], intention to quit smoking, and intention to increase physical activity, both measured on a 1-item 7-point Likert scale (1=I certainly plan to quit smoking/to be more physically active; 7=I certainly do not plan to quit smoking/to be more physically active).

#### Application Use

Application use was defined as use of the core intervention content (ie, the 6 components of both the smoking cessation and physical activity modules). Visits to the nontailored general information on the website were not counted. Three quantitative measures were used to assess application use. The first measure was initial use, defined as participants initiating the smoking cessation or physical activity module at least once. The second measure was prolonged use, defined as participants who completed at least one intervention component as part of the smoking cessation or physical activity module. The third measure was sustained use, defined as participants who completed at least one intervention component of the smoking cessation or physical activity module and then initiated either module at least once more later in the study. On the Web-based questionnaire, an option was provided to indicate that participants did not visit the website, so those who did not use or did not recall using the application could be excluded from further questions regarding satisfaction with the application. These participants received a question with predetermined response categories concerning their reasons for not using the modules (not enough time; because I live healthy; not necessary, because I think I am not at risk for or I do not have COPD; I wanted to visit the website, but I could not log in to the website; other reason). The perceived influence of updating and adding new information to the website and sending periodic email prompts on application use was examined qualitatively during the interviews.

#### Satisfaction With the Intervention Content

Quantitative measures for satisfaction with the application included 7 questions on a 5-point Likert scale, ranging from 1=totally disagree to 5=totally agree and 1 question on a 10-point scale. These questions were largely based on earlier work of de Vries et al [11]. The 5-point questions assessed appreciation of the website (navigation), the tailored feedback (comprehensibility, novelty, usefulness, and personalization), and the application in general (recommendable to others, intention for future use). The 10-point question assessed overall satisfaction with the tailored feedback (1=very bad to 10=very good). Satisfaction with the application was further explored qualitatively during the interviews using the above-described topics as lead questions. The Web-based questionnaire asked participants to comment on their opinion of the application.

### Data Analyses

#### Quantitative Analyses

Categorical variables were represented by number and percentage and numerical variables by mean and standard deviation (SD) or median and interquartile range (IQR). To determine whether selective missingness had occurred for the outcomes satisfaction with the application and physical activity, we conducted chi-square tests for categorical and independent samples *t* tests for numerical baseline characteristics. Differences between the 2 recruitment channels regarding study participation and retention of the overall sample (control and intervention group) were assessed with chi-square tests. All further analyses only included the intervention group. Differences between the 2 recruitment channels regarding baseline characteristics were analyzed with chi-square tests, Fisher’s exact tests, or Fisher-Freeman-Halton tests for categorical variables and independent samples *t* tests or Mann-Whitney U tests for numerical variables. Logistic regression analysis and linear mixed models analysis were performed to determine the influence of satisfaction on the primary outcomes of the RCT, which were 7-day point abstinence for smoking cessation (0=did not refrain from smoking during the last 7 days or 1=refrained from smoking during the last 7 days) and MET minutes per week for physical activity measured at baseline and after 6 months. Linear mixed models were used for the physical activity to account for the correlation between repeated measurements of the same participant. As for correction, the models included baseline variables that were related to missing data. Multiple logistic and linear regression analyses were performed to assess differences in use and satisfaction in the intervention group, respectively, according to participant characteristics. Initial, prolonged, and sustained use (0=no or 1=yes) were the dependent variables to assess differences in use. The dependent variable to assess differences in satisfaction was overall satisfaction rated on a scale of 1 to 10. The following participant characteristics were included as predictors in each model: age, gender, education level, COPD status, dyspnea status (coded as 0=participants who scored 1 to 5 on the MRC dyspnea score and 1=participants who indicated experiencing no breathlessness), smoking status, level of physical activity, and the intention to quit smoking or increase physical activity (whichever intention was the highest). Only participants who completed at least one intervention component and consequently had received tailored feedback were included in analyses concerning satisfaction with feedback. Independent variables were checked for multicollinearity, where a variance inflation factor of >10 indicates a collinearity problem [52]. Missing values were imputed for the level of physical activity (covariate) and the level of satisfaction (outcome variable). Multiple imputation technique was used separately for the logistic regression analyses concerning usage outcomes (initial, prolonged, and sustained use) and for the linear regression analyses concerning satisfaction, with each 100 imputations and 100 iterations, using all variables in the multiple regression model (outcome as well as independent variables) as predictors for the missing values. All statistical analyses were performed using SPSS version 19 (IBM Corp).

#### Qualitative Analyses

Field notes of communication between participants and the research team and comments in the Web-based questionnaire were reviewed. Interviews were transcribed verbatim and content analysis was performed using the constant comparative method [53]. Using open coding, descriptive codes were assigned, compared, and contrasted to simultaneously define and refine their properties, subcategories, and categories. Coding took place by 2 researchers (Viola Voncken-Brewster and Mylène Amoureus) independently. Analytical sessions were held after every 2 to 3 interviews, in which the 2 researchers discussed the codes and analyses. New interviews and analytical sessions were planned until consensus and data saturation were reached. The outcomes of these sessions were discussed with Huibert Tange, Trudy van der Weijden, and Hein de Vries in order to integrate these results with the quantitative results and aid understanding of the research problems.

## Results

### Participant Characteristics

**Figure 2:**
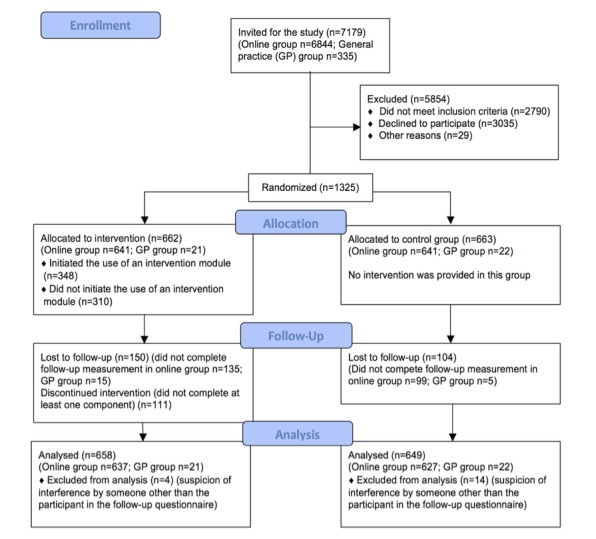
Consolidated Standards of Reporting Trials diagram, as shown in RCT results article [18].

A total of 1325 participants completed the baseline questionnaire and were randomly assigned to the intervention (n=662) and control group (n=663), of which 1307 (98.6%) participants were included in the analysis (658/662, 99.4%, in the intervention group and 649/663, 97.9%, in the control group). Figure 2 shows the Consolidated Standards of Reporting Trials diagram as shown in the RCT results article [18].

In the online group, 59.5% (4072/6844) of invited individuals completed the baseline questionnaire. In the general practice group, only 12.8% (43/335) of invited individuals completed the baseline questionnaire, which is significantly lower (*P*<.001). A priori, both groups differed in COPD status. In the online group, the COPD screening test (RHSQ) was part of the baseline questionnaire, and afterwards only 31.5% (1282/4072) were eligible for the study. A total of 18 participants of the online group were excluded from analyses due to a high level of suspicion of interference by someone other than the participant; consequently, 1264 participants were included in analyses. Interference was suspected when at least 2 of the following variables did not match their Flycatcher profile on the follow-up questionnaire: sex, day of birth, year of birth. If only one variable was inconsistent or day and month were reversed, we suspected a typing error and did not exclude those participants. The general practice group was already screened by their general practitioner, and only eligible patients received an invitation. Retention was higher in the online group (*P*<.001), where after excluding the 18 participants, 81.5% (1030/1264) of participants completed the follow-up questionnaire, compared to 53.5% (23/43) of participants in the general practice group.

Due to the low response rate in the general practice group, the reminder protocol was adjusted for this group. Participants who responded confirmative to the invitation or reminder received 2 additional reminders by email if they had not completed the baseline questionnaire. They also received 2 reminders instead of one for the follow-up questionnaire. Table 1 presents the baseline participant characteristics of the intervention group overall and the online panel and general practice group separately. The only significant differences between the 2 groups were educational level (*P*=.049) and intention to quit smoking (*P*=.01).

### Characteristics of Interviewees

The age of the 10 participants who were interviewed ranged from 42 to 69 (median 58) years. There were 5 male and 5 female participants, 3 were smokers and 7 did not smoke, 6 were diagnosed with COPD and 4 were at increased risk for COPD. Education level varied—3 had a high level, 4 an intermediate level, and 3 a low level of education. A total of 7 participants were from the online panel group and 3 from the general practice group.

**Table 1:** Baseline characteristics of participants of the intervention group—overall, general practice and online group.

Characteristic		Intervention group overall (n=658)	Intervention group general practice (n=21)	Intervention group online (n=637)	*P* value
Age, years, mean (SD)^a^		57.7 (7.3)	58.6 (8.6)	57.7 (7.3)	.55
Male, n (%)^b^		326 (49.5)	11 (52.4)	315 (49.5)	.79
**Education level, n (%)^b^**					.049
	Primary school/basic vocational school	191 (29.0)	10 (47.6)	181 (28.4)	
	Secondary vocational school/high school degree	209 (31.8)	2 (9.5)	207 (32.5)	
	Higher professional degree/university degree	258 (39.2)	9 (42.9)	249 (39.1)	
**COPD^d^ status, n (%)^c^**					.06
	Diagnosed with COPD	146 (22.2)	1 (4.8)	145 (22.8)	
	Increased risk for COPD per RHSQ^e^	512 (77.8)	20 (95.2)	492 (77.2)	
**MRC^f^ dyspnea (n=657)^g^, n (%)**					.11
	No breathlessness	177 (26.9)	8 (40.0)	169 (26.5)	
	1	264 (40.2)	9 (45.0)	255 (40.0)	
	2	167 (25.4)	1 (5.0)	166 (26.1)	
	3	34 (5.2)	1 (5.0)	33 (5.2)	
	4	9 (1.4)	1 (5.0)	8 (1.3)	
	5	6 (0.9)	0 (0.0)	6 (0.9)	
**Smoking status, n (%)^b^**					.09
	Currently smoking	241 (36.6)	4 (19.0)	237 (37.2)	
	Currently not smoking	417 (63.4)	17 (81.0)	400 (62.8)	
Intention to quit smoking (1=highest intention, 7=lowest intention) among smokers (n=241), median (IQR^h^)^i^		4.0 (2.0-5.5)	1.0 (1.0-1.8)	4.0 (2.0-6.0)	.01
Level of physical activity (MET^j^ per week) (n=555), median (IQR)^i^		2904.0 (1200.0-5758.0)	3036.0 (74.3-4518.8)	2904.0 (1212.-5787.5)	.36
Intention to be more physically active (1=highest intention, 7=lowest intention), median (IQR)^i^		3.0 (2.0-4.0)	3.0 (1.0-4.5)	3.0 (2.0-4.0)	.64

^a^Independent samples *t* test.

^b^Chi-square test.

^c^Fisher’s exact test.

^d^COPD: chronic obstructive pulmonary disease.

^e^RHSQ: Respiratory Health Screening Questionnaire.

^f^MRC: Medical Research Council.

^g^Fisher-Freeman-Halton test.

^h^IQR: interquartile range.

^i^Mann Whitney U test.

^j^MET: metabolic equivalent task.

### Application Use

#### Quantitative Results

##### Initial Use

A total of 52.9% (348/658) of the intervention group started at least one of the two modules, with an average of 2.0 (SD 2.1, range 1 to 20) initiations in this group. The smoking module was initiated by 33.2% (80/241) of smokers and the physical activity module by 44.7% (294/658) of participants (both smokers and nonsmokers). The smoking cessation module was also initiated by 7.2% (30/417) of nonsmokers. Participant characteristics did not predict initial use significantly (Table 2). Of the participants who initiated use, 23.6% (82/348) indicated at some point during the intervention period that they did not want to receive prompts any longer.

##### Prolonged Use

As described earlier [18], of all participants in the intervention group, 36.0% (237/658) completed at least one intervention component. This group completed on average 2.1 (SD 2.4, range 1 to 21) components. At least one component of the smoking cessation module was completed by 21.2% (51/241) of smokers, and 29.3% (193/658) of participants completed a component of the physical activity module. A total of 1.7% (7/417) of nonsmokers completed at least one component of the smoking cessation module. None of the participant characteristics were significant predictors of prolonged use (Table 2). Table 3 shows how often the individual components of each module were completed and the proportion of each completed component compared to the total number of all completed components in each module.

##### Sustained Use

A total of 16.6% (109/658) of participants revisited the intervention content. They initiated one of the two modules after they had finished an intervention component of either module earlier in the study. Older participants, those diagnosed with COPD, and participants who reported breathlessness (MRC score ≥1) were significantly more likely to revisit the intervention content (Table 2). For physical activity, 3.0% (20/658) of participants used the health risk appraisal component more than once. The goal setting and action planning component was completed several times by 2.3% (15/658) of participants. For smoking cessation, 0.8% (2/241) of smokers completed the health risk appraisal component multiple times, and 2.5% (6/241) of smokers completed the goal setting and action planning component more than once.

**Table 2:** Results of logistic and linear regression analyses of participant characteristics with initial use, prolonged use, sustained use, and satisfaction as dependent variables. VIF^a^≤1.32.

	Initial use (N=657)		Prolonged use (N=657)			Sustained use (N=657)			Satisfaction^b^ (N=237)	
	OR^c^ (95% CI^d^)	*P* value		OR (95% CI)	*P* value	OR (95% CI)	*P* value	Regression coefficient^e^ (95% CI)		*P* value
Gender (female vs male)	0.98 (0.83-1.15)	.89		1.19 (0.84-1.68)	.32	1.12 (0.90-1.41)	.61	–0.21 (–0.69 to 0.28)		.40
Age	1.01 (0.99-1.03)	.35		1.02 (0.99-1.04)	.18	1.04 (1.02-1.05)	.02	–0.02 (–0.05 to 0.02)		.38
COPD^f^ status (COPD vs at risk for COPD)	1.39 (0.96-2.02)	.10		1.38 (0.93-2.05)	.11	1.83 (1.15-2.91)	.01	–0.16 (–0.69 to 0.36)		.55
Education level (low vs high)	0.96 (0.64-1.42)	.82		0.71 (0.47-1.09)	.12	0.82 (0.47-1.44)	.48	0.56 (–0.07 to 1.18)		.08
Education level (medium vs high)	1.45 (0.982.1-4)	.06		1.29 (0.87-1.90)	.20	1.36 (0.83-2.22)	.23	0.40 (–0.15 to 0.96)		.16
Smoking status (smoking vs not smoking)	0.77 (0.56-1.06)	.13		0.76 (0.54-1.08)	.14	0.72 (0.57-0.92)	.18	–0.17 (–0.72 to 0.39)		.56
Level of physical activity (MET^g^ per week)	1.00 (1.00-1.00)	.69		1.00 (1.00-1.00)	.57	1.00 (1.00-1.00)	.88	0.00 (0.00-0.00)		.93
Intention change behavior (1=highest intention, 7=lowest intention)	0.93 (0.85-1.03)	.15		0.93 (0.84-1.03)	.14	0.89 (0.78-1.02)	.08	–0.18 (–0.32 to –0.03)		.02
Dyspnea status (no breathlessness vs breathlessness)	1.01 (0.70-1.45)	.97		0.71 (0.48-1.04)	.08	0.55 (0.31-0.96)	.04	–0.43 (–1.01 to 0.15)		.15

^a^VIF: variance inflation factor.

^b^Only participants who completed at least one intervention component were included in this analysis.

^c^OR: odds ratio.

^d^CI: confidence interval.

^e^Linear regression coefficient indicates the effect of this variable on satisfaction after correction for the other variables in the model.

^f^COPD: chronic obstructive pulmonary disease.

^g^MET: metabolic equivalent task.

**Table 3:** Total number of completed components for each module.

	Health risk appraisal n (%)	Motivational beliefs n (%)	Social influence n (%)	Goal setting and action planning n (%)	Self-efficacy to change or maintain behavior n (%)
Physical activity	141 (37.9)	64 (17.2)	14 (3.8)	86 (23.1)	67 (18.0)
Smoking	10 (8.1)	16 (12.9)	13 (10.5)	48 (38.7)	37 (29.8)

A total of 130 participants reported that they did not log in to the website. The following reasons for not using the website were given (some participants gave multiple reasons): 26.9% (35/130) of participants did not have enough time; 23.8% (31/130) found it not necessary because they lived healthy; 27.7% (36/130) did not think it was necessary because they thought they were not at risk for or did not have COPD; 9.2% (12/130) of participants wanted to visit the website but could not log in; 19.2% (25/130) of participants gave other reasons. For example, they forgot about the website or they felt too confronted or were not ready to change behavior.

#### Qualitative Results

The interviews revealed that adding new information to the website led to more application use. One interviewee indicated that he might have used the application more often if it would have been available as a smartphone application.

An app...stimulates you more to use it, because you walk around with that thing [smartphone] all the time.

Opinions about the periodic prompts to increase the use of the application were positive except for interviewees who were not satisfied with the application. One participant suggested sending prompts directly to his smartphone instead of through email to his computer. He also would have liked the possibility to change the prompt frequency to his preferences.

I think that you have to make the website that you can select yourself how often you want to receive an email prompt.

### Satisfaction

#### Quantitative Results

Selective missingness did not occur; we found no significant differences in characteristics of participants who completed the process evaluation questionnaire compared to participants who did not complete this questionnaire. Table 4 shows the results concerning satisfaction. A total of 80.1% (257/321) of participants who used the website agreed (ie, 4=agree or 5=totally agree on the Likert scale) that it was easy to navigate the website. In total, 78.9% (135/171) of participants who completed at least one intervention component thought the tailored feedback was clear, 23.2% (40/172) agreed that the messages contained new information, 39.5% (68/172) indicated that these helped them live healthier, and 32.2% (55/171) thought that the feedback was personally relevant. A total of 56.6% (193/341) of participants who visited the website would recommend the application to others, and 32.3% (108/334) would like to use it in the future. Participants who completed at least one intervention component gave the feedback an average score of 6.7 (SD 1.6) on a scale from 1 to 10. Satisfaction with the intervention content did not have a significant impact on the primary outcomes of the RCT for smoking cessation (OR 1.30, 95% CI –0.59 to 2.87, *P*=.51) and physical activity (estimated mean difference 0.20, 95% CI –329.77 to 403.02, *P*=.84). Regarding the influence of participant characteristics on satisfaction, multiple linear regression analysis showed that participants with greater intention to change behavior rated the tailored feedback higher (Table 2).

#### Qualitative Results

Overall, participants were satisfied with the usability of the website and clarity of the tailored feedback. However, some questions were perceived to be hard to answer. One interviewee indicated that this could result in misinterpretations, which would compromise the accuracy of the tailored feedback. All interviewees except for one suggested that the information given in the tailored feedback was mostly not new to them. However, some of the participants still found the information useful, since it gave good attainable advice, confirmed their knowledge, provided support, and prompted behavior change.

Yes, I already knew that...but yes, it provided support.

**Table 4:** Results of the satisfaction questionnaire.

	Mean (SD)	Answer categories (%)				
		1	2	3	4	5
It was easy to find information on the website (1=totally disagree, 5=totally agree), (n=321)^a^	4.0 (0.8)	4.4	6.5	9.0	50.8	29.3
The tailored feedback was clear (1=totally disagree, 5=totally agree), (n=171)^b^	4.0 (0.8)	2.3	1.2	17.5	56.7	22.2
The tailored feedback contained information that was new to me (1=totally disagree, 5=totally agree), (n=172)^b^	2.8 (1.0)	8.1	30.2	38.4	20.3	2.9
The tailored feedback helped me to live healthier (1=totally disagree, 5=totally agree), (n=172)^b^	3.1 (1.0)	8.7	14.5	37.2	34.9	4.7
The tailored feedback was personally relevant to me (1=totally disagree, 5=totally agree), (n=171)^b^	2.9 (1.0)	9.9	22.8	35.1	29.2	2.9
I would recommend MasterYourBreath to others (1=totally disagree, 5=totally agree), (n=341)^a^	3.5 (9.2)	3.8	7.3	32.3	46.0	10.6
I would like to use MasterYourBreath in the future (1=totally disagree, 5=totally agree), (n=334)^a^	3.0 (9.8)	9.0	18.0	40.7	29.0	3.3
Rating of tailored feedback on a scale of 1 to 10 (1=very bad, 10=very good), (n=182)^b^	6.7 (1.6)	—	—	—	—	—

^a^Only participants who indicated they visited the website were included in the analysis.

^b^Only participants who completed at least one intervention component were included in the analysis.

Reasons for finding the application not useful included already maintaining a healthy lifestyle, not noticing any progress or effect on health, not being able to decrease medication use, and not believing that a computer program can help change behavior.

Because I did not really notice any progress or anything, I was not very motivated to continue [MasterYourBreath]

Opinions about personalization of the tailored feedback were mixed. Suggestions were given such as focus more on comorbidities and rehabilitation therapy. Some participants indicated that the feedback was personal, and one participant mentioned that the feedback was equal to a health care professional’s advice. Advantages of using the computer were being able to access the application any time and receiving a good overview of the information, which made it easier to process and remember. On the other hand, it was often indicated that automated computer feedback could never be personal enough and that a conversation with a health care professional would be preferred or should be added to the intervention.

It’s hard to influence patients from a distance by computer...I’d rather talk 5 minutes to my general practitioner than sit behind a computer.

Interviewees would recommend the application especially to skilled computer users with an unhealthy lifestyle or lung complications. Interviewees who found the application useful indicated that they wanted to keep using it in the future, while others did not. One participant emailed the research team that the results of the RHSQ could scare people unnecessarily. It was also mentioned in the comment section of the Web-based questionnaire that it felt like COPD was imposed upon people.

It sometimes seemed like they want to talk you into having COPD.

## Discussion

### Principal Findings

This process evaluation explored application use and satisfaction with the MasterYourBreath intervention in order to uncover possible reasons for insufficient use of the application, which could partly explain the lack of treatment effect in the RCT [18]. Results showed that only half of the participants in the intervention group initiated one of the modules. In addition, participants did not use a significant part of the intervention content, as only 36.0% of participants completed at least one component and 16.6% revisited the intervention.

The RCT included the following evidence-based measures to promote application use: sending email prompts to participants [49]; updating and adding new information to the website regularly [46-48]; dividing the application into small components, since participants were apprehensive about the length of the application during the usability [24] and pilot study [26]; and including interactive behavior change strategies with multiple feedback moments, such as the possibility to monitor behavior change and track goal achievement over the course of the study [46,48]. Only participants who found the application helpful indicated that sending periodic email prompts and updating the website regularly were beneficial for increasing application use. A suggestion to increase use was to develop a MasterYourBreath application for smartphone and provide an option to select a prompt frequency to one’s preference. As described elsewhere [18], shortening the application by giving participants the opportunity to choose intervention components might have led to a decrease in application use as more freedom in navigation leads to less application use [54,55] and similar studies that directed participants through an intervention pathway yielded positive treatment results [11,17,35,36,56-58]. Monitoring progress was given as an option. Yet only a limited number of participants initiated these components, and few used these components multiple times. Future interventions may therefore provide this information as part of the standard feedback, because facilitating self-monitoring of behavior and progress toward goals have also been found to be powerful behavior change techniques [59]. Freedom of navigation could thus also be an explanation for why the components related to monitoring behavior and tracking goal attainment did not have the anticipated positive effect on application use. Directing participants through a specified intervention pathway might improve the use of these components. It was also interesting that participants chose the social influence component least of all components. We expected this, as results of the usability and pilot study showed that participants thought that the norms and behavior of others were irrelevant to the participants’ behavior change process [24,26]. Nevertheless, it is important to include a social support component and promote its use, since a meta-analyses of COPD smoking cessation interventions showed that “advice on/facilitate use of social support” was one of the few effective behavior change techniques [59]. Hence, the identification of strategies that increase the attractiveness of social support components for this group may be a first essential step toward promoting use of these components.

### Reasons for Low Application Use

Several causes for low application use were identified. First, examining the participant characteristics in relation to application use, we found that being diagnosed with COPD, experiencing breathlessness, or being of an older age was linked to revisiting the intervention content (sustained use). Meanwhile, over a quarter of the participants who did not use the application indicated that not being diagnosed with COPD and believing not to be at risk for COPD were the reasons for this. Participants who were not diagnosed with COPD, were of a younger age, and did not experience breathlessness might have dismissed the opportunity to use or revisit the application because they felt that the application was not relevant for them. However, the application could especially benefit these groups, since early smoking cessation is extremely important for achieving a better health status and improving life expectancy in individuals susceptible for airflow obstruction [60]. We suspect that the lower application use in these groups could have been caused by insufficient tailoring of the study invitation and application for participants who were younger, only at risk of COPD, and did not experience breathlessness. Although the main intervention content (the smoking cessation and physical activity module) was tailored to these groups, the study invitation and general information on the website were not. The overall focus of the information was on COPD; while this is relevant for COPD patients, it can be experienced negatively by others, as our qualitative data show. Instead, information should focus more on smoking cessation and changes in physical activity and their health benefits in general. The information on COPD and the link between lifestyle behaviors and COPD should still be provided, but this could be limited to a few sections. The importance of early smoking cessation should be emphasized in these groups so the relevance of the application will be more evident for participants who are younger, only at risk for COPD, and who do not experience breathlessness.

The second reason for low application use was that participants indicated that they did not need the application because they found that their lifestyle was healthy. However, our results based on smoking cessation and physical activity data did not confirm an influence of lifestyle on application use. It seems therefore that participants might have used their belief in a healthy lifestyle as an explanation for the lack of need for change. According to our data, 42% of the participants who indicated that their lifestyle was healthy smoked or did not adhere to the physically activity norm (defined as being physically active at least 5 days a week, 30 minutes a day at moderate or vigorous intensity). Participants received feedback regarding their smoking behavior and (non)adherence to the physical activity norm only when they used the application and completed the health risk appraisal component and not during the baseline measurement. Providing this feedback at baseline could have promoted use, as another study found that feedback regarding partial or nonadherence to lifestyle recommendations was positively associated with module use [61]. In contrast to our results, a study by Schneider et al [29] found that individuals with an unhealthy lifestyle were more likely to initiate the program but less likely to complete a module. These earlier studies [29,61] measured module use, while this study measured completion of components. This was inherent in the intervention design, as participants were not steered toward completing a module but were given the option to select components. An explanation for the difference in study results between our study and Schneider et al [29] could be differences in the intervention design. Future research is needed to uncover which design would be best to improve use among individuals with an unhealthy lifestyle.

A third reason for not using the application was experiencing problems logging in to the website. Taking into consideration participants might forget their account information, we provided a personalized link in the prompt emails to access the application without having to log in. The prompt emails containing the personalized link were sent once participants started using the application. This link should also have been embedded in the first invitation to access the website instead of the log-in information so participants never had to log in. However, the invitation email should emphasize that this is their personal account, as never using a password could make it difficult for participants to realize this.

### Satisfaction With the Intervention Content

Satisfaction with the intervention content did not have an effect on the primary outcomes of the RCT (ie, smoking cessation and physical activity). Satisfaction was higher among people with a greater intention to change their behavior. Other characteristics did not have a significant influence on satisfaction. In this study, the intervention content was tailored to user’s preference [10], and participants were free to choose which intervention components they wanted to complete. Tailoring the use of components to the level of motivation to change their behavior may be helpful for future interventions to increase satisfaction among those with a low intention to change. When dividing satisfaction into different categories, we found that participants rated navigation and comprehensibility the highest. We expected these results, since these aspects were improved during the usability evaluation [24]. Novelty of the tailored feedback scored lowest, but although the information was not new to the participants, it was often still considered to be useful to support healthy living. Reasons for not finding the application useful were not seeing any progress in managing the disease, generally not believing that a computer program could help them, or already maintaining a healthy lifestyle. Yet the qualitative results did not confirm that participants who maintained a healthy lifestyle were less satisfied with the intervention. Even though the feedback was tailored to participant needs, personal relevance scored relatively low. A suggestion was to focus more on comorbidities and rehabilitation therapy, while it was also suggested that a computer could not provide the level of personalization that a health care provider could. Involvement of a health care provider might not only increase satisfaction but also application use [47]. A study by Tabak et al [62] also suggests that the involvement of a health care provider plays an important role in COPD patient adherence to a telehealth intervention, since the modules with low involvement by a health care professional were accessed considerably less often. Another study found similar results: the use of a COPD Web-based self-management platform was higher when the platform was integrated into a disease management approach with trained health care providers encouraging patients to use the platform and when most substantial personal assistance was provided by a research nurse [63]. Our pilot study [26], which included the support of a practice nurse, also showed a considerably higher number of revisits compared to this study. Originally we planned to involve practice nurses in this study; however, due to recruitment problems we could not integrate the application into primary care, which made it unfeasible to involve practice nurses.

### Recruitment in the General Practice Setting

This process evaluation confirms that recruiting participants for this study is relatively difficult in a general practice setting. The participation rate and retention rate were significantly lower in the general practice group compared to the online group, despite of extra reminder efforts that had been made in the former group. This is in line with results of other online behavior change intervention studies that included an average of approximately 1 participant per practice in one study [64] and 5 participants per practice in another study [65] compared to approximately 9 participants recruited per practice in this study. These studies also found that recruitment via general practices tends to be less cost effective and yields a lower net effect compared to the Internet, newspaper, and other channels [64,65]. A partial explanation for the relatively positive results in the online group could be that these individuals had been more motivated to complete questionnaires, since they signed up to be a member of a company for online research and expected to receive research questionnaires for which they received a small reimbursement [66]. Moreover, they all had access to a computer with Internet and received the study invitation through email. It was not possible to invite patients of the general practice group through email, which posed an additional barrier for patients to start the study, as they had to transfer from reading the invitation letter to signing up for the study online.

To address recruitment issues in general practice, a different study design might be helpful. Nagykaldi et al [67] propose a design that involves an implementation phase before the start of the study. During this phase, integration of technology into the delivery of usual care can be accomplished as part of practice improvement, after which a subset of patients can be invited for study participation. This approach might motivate practices and patients to participate, as it focuses on patient health and practice improvement as well as research. Another strategy that could be explored to improve patient recruitment is the use of practice facilitators [68]. Practice facilitators are trained health care professionals, and their main tasks are to assist practices in research and quality improvement projects. Their work includes building long-term relationships with practices, improving communication, and facilitating system-level changes [68]. Practice facilitators can help successfully implement an eHealth intervention in primary care, serving as a resource for practices while aiding the practice level implementation phase of research projects. Sustaining the work of practice facilitators would require structural financial resources. A review of the practice facilitation literature showed that practice facilitators are usually hired by health care authorities, academic medical centers, or through funding from academic research grants [69]. More research is necessary to uncover optimal recruitment strategies of participants in a primary care setting.

### Limitations

This study has several limitations. First, participants were mainly recruited through an online panel, which could decrease the external validity of the presented results, as recruitment channel may impact the type of individuals participating in the study. For instance, a study found that recruitment via general practices resulted in a larger proportion of lower educated smokers and COPD patients compared to mass media recruitment [64]. Second, interviewing only participants who used the application might have led to limited information on why participants did not use the application. While reasons for not using the application were revealed by interviewees who used the application minimally, these might differ from participants who did not use the application at all. Third, we did not ask participants what kind of help they would have appreciated from a health care provider and how the application could provide a supporting role when working with a health care provider. This could have provided more insight to which elements of self-management support can be offered effectively by a computer application and when personal support would be needed. Future research should focus on how technology can be effectively integrated into and leveraged in a primary care setting.

### Conclusion

This process evaluation revealed several potential causes for the insufficient use of a Web-based COPD self-management application. Although believing that they lived a healthy lifestyle was for certain individuals a reason to not use the program, on a group level lifestyle did not seem to influence application use. Older individuals, those diagnosed with COPD, and those who experienced breathlessness were more likely to revisit the application. To improve application use among younger participants, those at risk for COPD, and those who do not experience breathlessness, we recommend emphasizing the importance of early smoking cessation for health benefit. In addition, we recommend focusing less on COPD and more on general health benefits of changing lifestyle behaviors for the group that is only at risk for COPD. Involvement of a health care professional could improve participant satisfaction with the intervention and may increase engagement with the intervention materials. However, participation and retention rates in the general practice group were low, and online recruitment limits the possibilities of integrating involvement of a health care professional. We suggest that, in order to improve study participant recruitment rates in general practices, technology is integrated into the practice workflow prior to the start of the study and practice facilitators are used to accelerate the implementation process.

## Abbreviations

COPD: chronic obstructive pulmonary disease
IPAQ-SF: International Physical Activity Questionnaire–Short Form
IQR: interquartile range
MET: metabolic equivalent task
MRC: Medical Research Council
RCT: randomized controlled trial
RHSQ: Respiratory Health Screening Questionnaire
